# Toxicity Assay and Pathogenic Process Analysis of *Clonostachys rogersoniana* Infecting *Cephalcia chuxiongica*

**DOI:** 10.3390/microorganisms13040709

**Published:** 2025-03-21

**Authors:** Junjia Lu, Jian Liu, Huali Li, Yajiao Sun, Shuwen Liu, Mengyao Wang, Yonghe Li

**Affiliations:** 1 College of Landscape Architecture and Horticulture Sciences, Southwest Forestry University, Kunming 650224, China; lujunjia@swfu.edu.cn (J.L.); jian927520@163.com (J.L.);; 2 Key Laboratory of Forest Disaster Warning and Control of Yunnan Province, Southwest Forestry University, Kunming 650224, China; 3 College of Plant Protection, Yunnan Agricultural University, Kunming 650201, China

**Keywords:** biological control, *Cephalcia chuxiongica*, *Clonostachys rogersoniana*, novel entomopathogenic fungi, media optimization, scanning electron microscopy

## Abstract

*Cephalcia chuxiongica* has caused significant damage to pine forests, becoming a major biological disaster that hinders the sustainable development of forestry in China. To investigate the efficacy of biological control measures, entomopathogenic fungi were isolated and purified from the larvae of *Ce. chuxiongica* that had succumbed to diseases. The pathogenic capacity of strains was assessed using bioassay methods, and their infection process was observed using scanning electron microscopy. ITS, LSU, and TEF analysis disclosed *Clonostachys rogersoniana* as the highly virulent strain responsible for the death of *Ce. chuxiongica*. The optimal medium for its mycelial growth and sporulation was found to be PPDA. In addition, the bioassay revealed that the median lethal time (LT_50_) for *Ce. chuxiongica* was 24.34 h and median lethal concentration (LC_50_) was 2.35 × 10^5^ conidia/mL, indicating that *C. rogersoniana* possesses potent virulence and demonstrates rapid pathogenicity. Furthermore, scanning electron microscopy demonstrated that *C. rogersoniana* initially entered the body of *Ce. chuxiongica* through the spiracle and progressively made its way into the body wall, resulting in the insect’s death. The mode of infection for *C. rogersoniana* is exceedingly rare. As a consequence, the results of this study can serve as a reference for the management of chewing insects, such as *Ce. chuxiongica*.

## 1. Introduction

**Figure 1:**
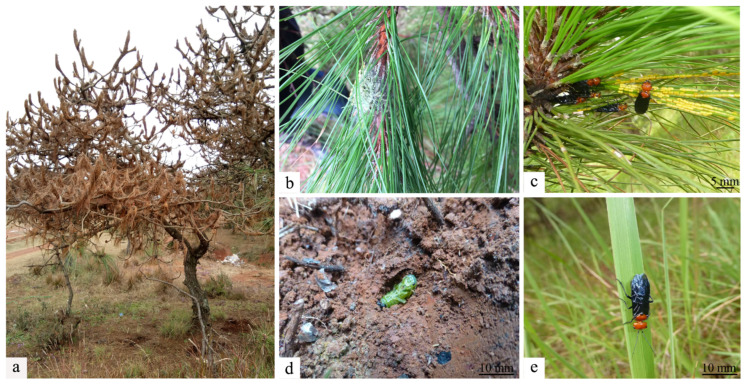
Morphological illustrations of insects. (**a**) Pine trees eroded by *Cephalcia chuxiongica*. (**b**) *Cephalcia chuxiongica* forms insect packets on trees after spinning silk. (**c**–**e**) *Cephalcia chuxiongica* lays eggs on pine needles and a diapause larva hides in a soil chamber.

The pine tree, a critical species in China’s extensive forest resources, is a highly adaptable and widely distributed species that embodies a variety of economic values. It has become an indispensable component of China’s economic system and operates in industries including pharmaceuticals, forest products chemistry, tourism, and construction [1,2,3]. *Cephalcia chuxiongica* primarily damages the needles of Pinaceae plants, including *Pinus yunnanensis*, *P. armandii*, and *Keteleeria evelyniana*, during its larval stage [4,5]. The mature larvae of *Ce. chuxiongica* directly feed on the pine needles. They have a voracious appetite and feed while concealed within their nest tents (Figure 1b). As the pine needles are consumed, the insect bags on the branches and shoots turn yellow, making the pine shoots appear scorched from a distance (Figure 1a). In late October, the mature larvae cease feeding, fall to the ground, and enter dormancy (Figure 1d), and these larvae can remain in diapause for over 22 months [6,7]. Once the larvae of *Ce. chuxiongica* metamorphose into imagoes, their remarkable flight capabilities render them all but impossible to capture. With ease, these imagoes initiate a new cycle of reproduction, inflicting further damage on pine forests (Figure 1c,e). These traits present significant challenges to pest control efforts. As a result, the species has caused extensive damage to the growth and development of coniferous forests in various regions, emerging as a major biological threat that severely impedes the sustainable development of forestry in China [5,8].

At present, the administration of *Ce. chuxiongica* is primarily reliant on the use of chemically synthesized pesticides, for example, Bifenthrin [9] and Lambda-cyhalothrin [10]. Nevertheless, the excessive and prolonged use of chemical pesticides results in a multitude of intricate problems. The primary issue was the evolution of pest resistance to these chemical agents and the accumulation of residues that exceeded regulatory thresholds. In recent years, research has shown that biological control, a cutting-edge pest management approach, plays a crucial role in restoring natural balance, effectively safeguards non-target organisms, and successfully circumvents the problems associated with chemical pesticides, making it a top choice in modern pest control. Currently, biological control chiefly depends on the pathogenic capacity of entomopathogenic fungi (EPF) against pests. The penetration stage of the EPF’s infection process commences when fungal propagules contact the host cuticle, and the fungi then employ their enzymes or toxins to assault the host through vegetative growth, resulting in damage or death [11,12]. For example, wireworms are capable of preying on *Ce. chuxiongica* larvae that are in a state of diapause within the soil environment. The larvae of *Ce. chuxiongica* can be parasitized by parasitic natural enemies from the family Ichneumonidae, which results in their death. Guesmi-Jouini et al. [13] conducted research and found that *Beauveria brongniartii* and *Bionectria ochroleuca* have the potential to infect the artichoke pest, resulting in its eventual death.

To date, research on the biological control of *Ce. chuxiongica* through the use of EPF has been exceedingly scarce. In light of the significant damage caused by this pest to pine forests and the limited implementation of biological control approaches in China at that time, the research was focused on the isolation of EPF from *Ce. chuxiongica* larvae that had naturally contracted disease. Employing morphological and molecular biological approaches, we successfully identified a strain demonstrating a high lethality rate against *Ce. chuxiongica*. A comprehensive evaluation of its growth characteristics was systematically carried out. Subsequently, a scanning electron microscope was utilized to meticulously examine the colonization process of the strain, and the median lethal time (LT_50_) and median lethal concentration (LC_50_) of the strain with respect to *Ce. chuxiongica* were accurately determined. These experimental results enabled us to elucidate the specific process and mode of infection of the strain in *Ce. chuxiongica*, thereby further assessing the pathogenicity of the fungus to *Ce. chuxiongica*. This study aims to provide a robust and reliable theoretical basis for formulating scientific, efficient, and highly targeted prevention and control strategies and methods for *Ce. chuxiongica*. Meanwhile, it is expected that the research outcomes will offer crucial references for the sustainable pest management strategies in the pine forests of this region.

## 2. Materials and Methods

### 2.1. Materials

From January 2023 to September 2024, larvae and imagoes of *Ce. chuxiongica* that died due to natural diseases were collected in Xundian County, Kunming City, Yunnan Province (25°31′ N, 102°56′ E, altitude 2100 m). They were transported to the laboratory right away for isolation and purification of pathogens after being refrigerated in a constant-temperature incubator at 4 °C. Simultaneously, healthy larvae in the diapause stage were collected, deposited in soil with a specific humidity level, and brought back to the laboratory for subsequent experiments.

### 2.2. Isolation and Purification of Pathogen

The cadavers of *Ce. chuxiongica* larvae were first rinsed with sterile water to eliminate any dirt residues. Subsequently, they were disinfected for 30 s in a solution consisting of 75% alcohol and 1% sodium hypochlorite [14]. After disinfection, the larvae were rinsed three times with sterile water. Excess moisture was then blotted dry using aseptic filter paper to ensure that the isolated fungi originated from *Ce. chuxiongica*. Under sterile conditions, we separated a portion of the insect tissue that had been infected with Swfuy-02 and placed it onto a Potato Dextrose Agar medium (PDA) (Table S1). To serve as a control group, the last rinse of sterile water was spread on a separate PDA to confirm that the larvae were thoroughly sterilized and that the isolated fungus was indeed the desired species [15]. A temperature-controlled incubator was used to cultivate the mycelia at a consistent temperature of 28 °C. After five days, the hyphae were transferred to a freshly prepared PDA. The purification process was subsequently administered consistently until a stable single colony was achieved, at which point the strain was designated Swfuy-02.

### 2.3. Pathogenicity Tests

The PDA medium was used to cultivate the Swfuy-02 strain for a duration of 20 days. The conidia that were adhered to the medium were subsequently meticulously rinsed off with a sterile aqueous solution containing 0.05% Tween-80. The resulting solution was subsequently centrifuged at a rotational speed of 5000 rpm for 20 min [16]. This centrifugation process effectively separated and removed the mycelium, obtaining a conidia suspension. Finally, the concentration of the conidia suspension was precisely adjusted to 1 × 10^8^ conidia/mL using a hemocytometer for subsequent experimental use [17].

The immersion method was employed to determine pathogenicity in accordance with Koch’s rule [18]. The larvae of *Ce. chuxiongica* were immersed in conidia suspension for a duration of 30 to 60 s. Each experimental group consisted of 30 larvae, and the entire process was repeated three times. Meanwhile, the control group was implemented through immersing the larvae in sterile water [19,20]. Every 6 h, the mortality rate, corrected mortality rate, and rate of stiffened larvae infected by Swfuy-02 were calculated by observing whether the fungus had successfully infected larvae and exhibited distinct symptoms, such as a powdery white substance on the surface or the larvae’s death.

The diseased larvae underwent further isolation and purification to obtain the strains. The strains were then inoculated onto healthy larvae to conduct re-infection tests. Larvae that exhibited symptoms of secondary infection were subsequently inoculated onto the PDA medium for isolation and purification purposes. The purification process was reiterated until single colonies were successfully acquired. Ultimately, the morphological characteristics of the strains in the pre-isolation and post-isolation states were compared. In the event that the morphology of the strain on the PDA medium was congruent with that of the original strain, it served as confirmation that this strain was the pathogen accountable for the mortality of *Ce. chuxiongica* [21,22,23]. The pathogenicity test was repeated 3 times.

(1) $$MR=\frac{N_{d}}{N}\times 100,$$

MR represents the mortality rate, Nd represents the number of larvae that die, and N represents the number of experimental samples.

(2) $$CMR=\frac{R_{t}-R_{c}}{1-R_{c}}\times 100\%,$$

CMR represents the corrected mortality rate; Rt and Rc represent mortality in the treatment group and control group, respectively.

(3) $$SR=\frac{N_{s}}{N}\times 100\%,$$

SR represents the rate of stiffened larvae, N_s_ represents the number of stiffened larvae, and N was the same as (1).

### 2.4. Morphological Identification and Molecular Phylogenetic Analysis

Swfuy-02 was morphologically identified following the method outlined by Raja H. A. et al. [24]. The primary culture medium for mycelium and sporulation growth was Czapek-Dox Agar medium (CZA) (Table S1), which was poured into a sterile Petri dish to a depth of approximately 1 mm. Five days later, the culture was removed for observation, and photographs were taken for documentation. Conidia images were captured by the Motic bio-optic microscope (MOTIC CHINA GROUP CO., LTD. in Xiamen, Fujian, China), which has a 20-megapixel fixed resolution and a 5-megapixel dynamic resolution and uses USB 2.0 for signal transmission.

The genomic DNA of the disease pathogen was extracted using the protocol outlined in the EZUP Column Genomic DNA Extraction Kit (Sangon Biotech (Shanghai) Co., Ltd. in Shanghai, China). The product was amplified using PCR with primers ITS, LSU, and TEF targeting sequences [25,26,27] (Table S2). The PCR was performed in a 25 μL reaction mixture following these specific conditions: an initial denaturation step was carried out at 95 °C for 5 min. This step aimed to fully separate the two strands of the template DNA, thus generating single-stranded DNA. Subsequently, 35 cycles were executed. Each cycle consisted of three main phases: denaturation at 94 °C for 30 s, during which the double-stranded DNA separated again; annealing at 52 °C for 45 s, allowing the primers to bind to the single-stranded DNA; and an initial extension at 72 °C for 50 s, with the goal of rapidly accumulating a large number of DNA fragments. Finally, a final extension step was performed at 72 °C for 10 min, which was intended to obtain complete and stable DNA fragments [28].

Subsequent analysis of the amplified product by agarose gel electrophoresis (1.5%) was carried out. Lastly, the purified and recovered product was sent to Beijing Tsingke Biotech Corporation (Beijing, China) for sequencing. The sequences obtained from Sanger sequencing were sent to NCBI’s GenBank database for BLAST comparison (https://www.ncbi.nlm.nih.gov/, National Center for Biotechnology Information) (accessed on 11 January 2025). The sequences of strains with significant homology were selected to construct a phylogenetic tree with the Neighborhood-Joining method of Mega 11.0. Then, the tree was evaluated by bootstrapping with 1000 replications.

### 2.5. Growth and Conidia Condition of Swfuy-02 on Different Medium

To investigate the impact of different media on the mycelial growth and sporulation of the Swfuy-02 strain, we selected PDA, CZA, Sabouraud Dextrose Agar with Yeast Extract medium (SDAY), and Peptone Potato Dextrose Agar medium (PPDA) [29] (Table S1). We selected the Swfuy-02 cake, which had been cultured on a PDA for more than 15 days and whose colony had fully covered the plate. We utilized a puncher to obtain a fungal plug with a diameter of 6 mm from the colony’s edge and inoculated it onto the plates of the aforementioned four media. The plates were incubated inverted in a constant-temperature incubator at 28 °C, with three replicates for each treatment. After 15 days of incubation, we employed the cross method to measure the colony’s diameter and analyzed the mycelial growth on various media. Once the mycelial growth rate measurement was completed, a 6 mm diameter puncher was employed to create holes halfway between the center and the edge of the colony. The fungal plugs were transferred to a centrifuge tube. Then, 10 mL of sterile water containing 0.05% Tween-80 was added. After vigorous shaking, the conidia count was conducted using a hemocytometer, and the resulting count was converted into conidia production per unit area [30,31].

### 2.6. Scanning Electron Microscopic Observation of the Body Wall of Swfuy-02 Infected Larvae

Samples were prepared by collecting the entire larvae. After being fixed in 3% glutaraldehyde overnight, the samples were rinsed three times with Phosphate-Buffered Saline (PBS). They were post-fixed with 1% osmium tetroxide for 1 h and subsequently rinsed three times with a PBS. A dehydration process was conducted in a series of concentrations, with each treatment lasting 25 min: 30%, 50%, 70%, 80%, 90%, 95%, and 100% ethanol [32]. For a duration of 1.5 h, the samples were infiltrated with tert-butanol and then freeze dried using the Hitachi-2030 freeze dryer. The Hitachi-1010 coater was employed to adhere and sputter the dried samples with molybdenum–palladium alloy. The Hitachis-3000n scanning electron microscope was employed to observe and photograph the prepared samples. The instruments mentioned above were all produced by Hitachi Group, which is located in Tokyo, Japan.

### 2.7. Swfuy-02 Effective Concentration Determination

To treat the larvae of *Ce. chuxiongica*, the concentration gradients of the conidia suspension of Swfuy-02 were specifically established at 1 × 10^4^, 1 × 10^5^, 1 × 10^6^, 1 × 10^7^, and 1 × 10^8^ conidia/mL, respectively. The experimental groups were composed of 30 larvae each, and three replicates were conducted. The method of inoculating the larvae with Swfuy-02 and the observational procedures were identical to those that had been previously described. Subsequently, the number of deceased larvae of *Ce. chuxiongica* under each treatment condition was tallied, and the mortality rate and corrected mortality rate of the larvae infected by Swfuy-02 were calculated. Then, the median lethal time (LT_50_) and median lethal concentration (LC_50_) of Swfuy-02 against *Ce. chuxiongica* were determined in accordance with the maximum lethal time (60 h) and the maximum lethal concentration (1 × 10^8^ conidia/mL) [33,34]. The pathogenicity test was repeated 3 times.

### 2.8. Statistical Analysis

The data were conducted with statistical software Graphpad Prism 9.5 and SPSS 26.0. Two-way ANOVA was used to evaluate the statistical significance of each sample (*p* < 0.05), and then Duncan’s multi-range test was carried out. Data were visualized using Adobe Photoshop 2023 and Adobe Illustrator 2020.

## 3. Results

### 3.1. Isolation of Strains Determination of Pathogenicity Analysis

**Figure 2:**
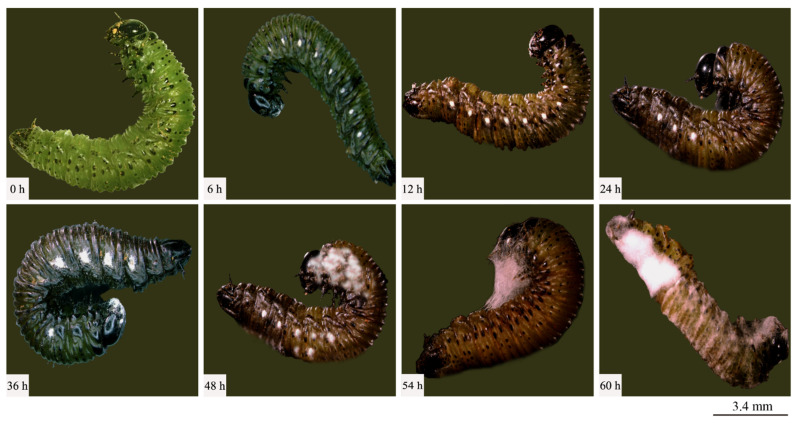
Symptoms of *Cephalcia chuxiongica* at different time points post-infection by *Clonostachys rogersoniana*.

A strain designated Swfuy-02 was isolated from the larvae of *Ce. chuxiongica* that had died from natural diseases in Xundian County, Kunming City, Yunnan Province. The Swfuy-02 strain was then re-inoculated onto the surface of *Ce. chuxiongica* larvae, with observations conducted at 6 h intervals (Figure 2). Six hours post-inoculation, the mycelium of the Swfuy-02 strain had progressively infiltrated the larvae’s bodies. Of the 30 larvae in the treatment group, six died, resulting in a corrected mortality rate of 17.78%. At 36 h post-inoculation, the number of dead larvae in the treatment group increased to 19, corresponding to a corrected mortality rate of 58.78%. By 60 h post-inoculation, almost 28 larvae in the treatment group had died, bringing the corrected mortality rate to 92.36%.

**Figure 3:**
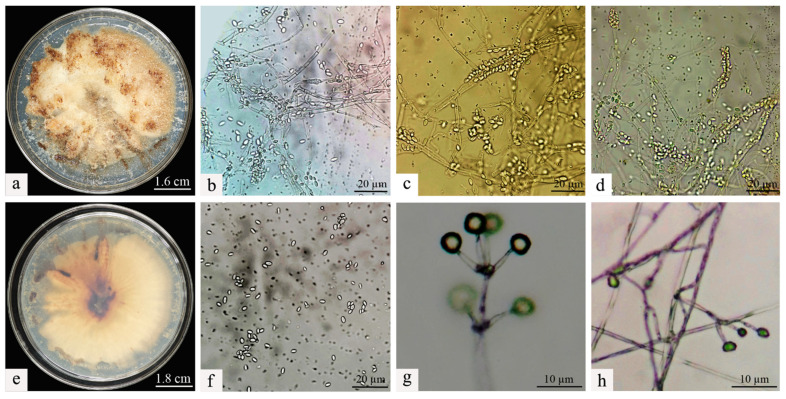
Morphological characteristics of *Clonostachys rogersoniana*. (**a**) Mycelial morphology of *Clonostachys rogersoniana* on Potato Dextrose Agar medium. (**e**) Abaxial mycelial morphology of *Clonostachys rogersoniana* on Potato Dextrose Agar medium. (**b**–**d**,**f**) Conidial morphology of *Clonostachys rogersoniana*. (**g**,**h**) Conidiophores that produce conidia.

The isolation and purification procedures were continued on the deceased larvae, and through repeated subculturing, stable single colonies were obtained. A comparison of the morphological traits of the two colonies was then conducted. It was observed that the morphological characteristics of the colonies on the PDA remained consistent before and after re-isolation. Specifically, the Swfuy-02 strain displayed a yellow-brown color on the PDA medium and produced brownish metabolic byproducts (Figure 3a,e).

### 3.2. Identification of Pathogen Analysis

The colonies of Swfuy-02 have a flesh-colored appearance and exhibit a flat morphology. They were characterized by numerous accumulations that resemble flesh-colored powder, along with indistinct, ring-shaped patterns. On the reverse side of the colonies, dark, wheel-like patterns and lumpy formations were visible.

The conidia of Swfuy-02 aggregate to form a head-like structure. Phialides were produced through the endogenous blastic phialidic (eb-ph) method. The branching points of the verticils show slight swelling, and the hyphae have the ability to aggregate into bundles. At the tips of the sterigmata, the conidia were unicellular and colorless. The dimensions of the sterigmata are 16 (−30) × 3.0 (−4.7) μm; the phialides measure 15.0 (−20.0) × 2.7 (−4.6) μm; and the conidia range from 5.0–7.5 × 2.5–3.0 (−3.5) μm. Additionally, structures resembling chlamydospores were present in the hyphae, with an approximate size of 24.5 × 14.7 μm (Figure 2). Based on morphological characteristics and comparisons with previous studies, the fungal isolate Swfuy-02 was identified as *Clonostachys* sp. (*Bionectriaceae*, Hypocreales, Ascomycota) [35,36].

**Figure 4:**
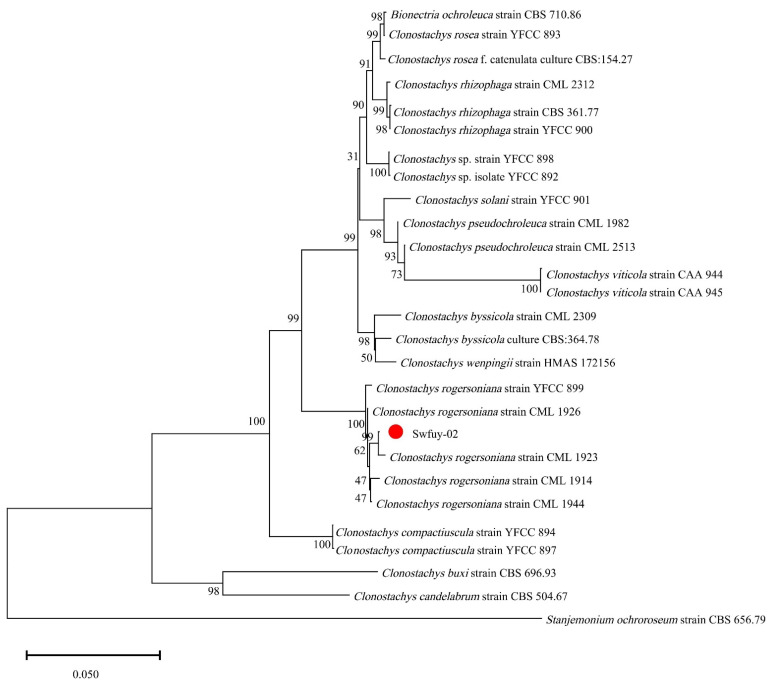
A phylogenetic tree of *Clonostachys rogersoniana* was generated using Neighbor-Joining methods of the combined ITS, LSU, and TEF. Scale bar = 0.05, which indicated that the average nucleotide difference between two sequences with a branch length of 1 unit was 0.050, meaning that approximately 5 out of every 100 nucleotide sites were different.

The pathogen’s Sanger sequencing results were compared using BLAST against the GenBank database at NCBI to assess their genetic relationships. Sequences showing significant homology were selected to construct the phylogenetic tree. As shown in Figure 4, Swfuy-02 clustered in a minor branch with *Clonostachys rogersoniana* (KC806291 of ITS, KX185019 of TEF, PP355367 of LSU), with a 99% support rate.

The results showed that Swfuy-02 was identified as *C. rogersoniana* by combining morphology and DNA sequencing.

### 3.3. Growth and Conidia Condition of Swfuy-02 on Different Media Analysis

After culturing the Swfuy-02 strain on Potato Dextrose Agar medium (PDA), Peptone Potato Dextrose Agar medium (PPDA), Czapek-Dox Agar medium (CZA), and Sabouraud Dextrose Agar with Yeast Extract medium (SDAY) for 15 days, notable differences in growth performance and sporulation characteristics were observed across the media. The strain exhibited superior growth, with both larger colony diameter and higher sporulation on PPDA medium. The colony diameter reached 83.62 ± 0.97 mm, and the sporulation quantity was 19.07 ± 0.18 × 10^6^ conidia/cm^2^, both significantly higher than those observed on the other media. On PDA and SDAY media, the colony diameters showed no significant difference, measuring 70.17 ± 1.2 mm and 71.22 ± 1.03 mm, respectively. However, the sporulation rates differed significantly: the strain produced 3.10 ± 0.16 × 10^6^ conidia/cm^2^ on PDA, while no sporulation occurred on SDAY medium. In contrast, the Swfuy-02 strain on CZA medium had a colony diameter of 63.67 ± 1.01 mm, significantly smaller than on the other media, and a sporulation quantity of 1.24 ± 0.12 × 10^6^ conidia/cm^2^, also lower than that on PPDA and PDA media (Table 1).

### 3.4. Scanning Electron Microscopic Observation

**Figure 5:**
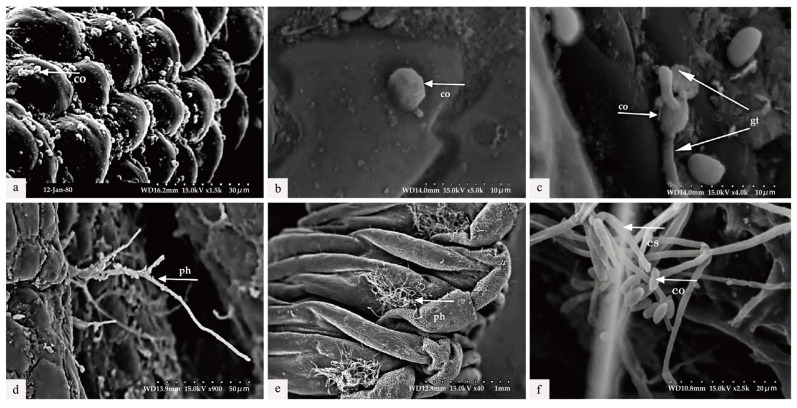
Scanning electron microscopic observation. (**a**) The conidia (co) were attached to the body wall (6 h) of the larvae of *Cephalcia chuxiongica*. (**b**) Penetration of conidia into the larval body wall (24 h post-inoculation). (**c**) The germination of appressoria into germ tubes (gt) (30 h following inoculation). (**d**) Certain hyphomycetes (ph) penetrated through the larval body wall (36 h post-inoculation). (**e**) Hyphae elongated (42 h post-inoculation). (**f**) The formation of a new conidiogenous structure (cs) and conidia was initiated (56 h post-inoculation).

The diseased larvae, treated at different time points, were collected for scanning electron microscope observation. Six h after treatment, a large number of conidia were observed successfully attaching to various areas of the body wall, including the spiracles and intersegmental membranes (Figure 5a). By 30 h post-inoculation, conidia germination was evident, with germ tubes emerging and penetrating the body wall of the larvae (Figure 5b,c). As shown in Figure 5c, the length of the germ tube had already surpassed half the diameter of the conidia. At 36 h post-inoculation, Swfuy-02 mycelium had directly penetrated the body wall and spiracles of *Ce. chuxiongica* larvae, entering the insect’s body (Figure 5d). At 42 h, the mycelium had increased around the spiracles of the body wall (Figure 5e). By 56 h post-inoculation, sporulation structures of Swfuy-02 were visible on the larvae’s body surface, with new conidia already formed (Figure 5f). Additionally, it can be observed from Figure 5 that the mycelium of Swfuy-02 initially invaded through the spiracles of *Ce. chuxiongica* larvae, whereas most other strains typically infect larvae through areas such as the head, tail, and intersegmental folds.

### 3.5. Swfuy-02 Effective Concentration Determination Analysis

**Figure 6:**
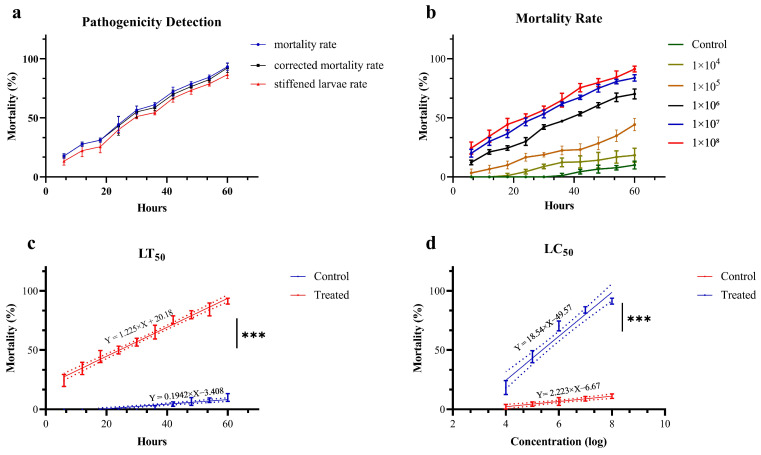
Pathogenicity of *Clonostachys rogersoniana* to *Cephalcia chuxiongica*. (**a**) Pathogenic determination of *Cephalcia chuxiongica* by *Clonostachys rogersoniana*. (**b**) The mortality rate of *Cephalcia chuxiongica* treated with conidia suspensions of *Clonostachys rogersoniana* at different concentrations. (**c**,**d**) Analysis of the regression equation for *Clonostachys rogersoniana* causing the death of half of the *Cephalcia chuxiongica* population. Error bars indicate the error range of the data. Shorter error bars represent smaller errors among the data points. Asterisks denote statistical significance, with *** corresponding to *p* < 0.001, indicating an extremely significant difference. The dash line illustrates the distribution pattern of the data points. A higher degree of polymerization indicates a stronger goodness of fit in figures.

*Ce. chuxiongica* was immersed with conidia suspension solutions of varying concentrations, and the mortality rate was recorded at 6 h intervals. As shown in Figure 6b, the lethal effect of Swfuy-02 on *Ce. chuxiongica* increased in tandem with the concentration of conidia. After applying a conidia suspension solution at a concentration of 1 × 10^8^ conidia/mL for 60 h, the mortality rate of *Ce. chuxiongica* reached 92.22%. A regression equation was then derived from the mortality data collected at the maximum observation time and the highest lethal concentration. The regression equation relating time to the mortality rate of *Ce. chuxiongica* was given by Y = 1.225X + 20.18, with the median lethal time (LT_50_) determined to be 24.34 h (Figure 6c). Furthermore, the regression equation relating conidia suspension concentration to the mortality rate of *Ce. chuxiongica* was Y = 18.54X − 49.57, and the median lethal concentration (LC_50_) was calculated to be 2.35 × 10^5^ conidia/mL (Figure 6d).

## 4. Discussion

### 4.1. Comparative Analysis with Preceding Research

The control of *Ce. chuxiongica* presents several challenges. Recent studies have highlighted the effectiveness of using entomopathogenic fungi (EPF) to target larvae during the diapause period [37,38,39,40]. In this study, a strain with strong pathogenicity to *Ce. chuxiongica* larvae was identified as *C. rogersoniana* through bioassays. First isolated from the rhizosphere soil of *Allium tuberosum* in 2008 [41], *C. rogersoniana* is now found in various environments, including the bark of dead twigs, soil, leaf litter, ascidians, and insects [42]. Our findings confirm that *C. rogersoniana* can parasitize *Ce. chuxiongica*. However, research on the entomopathogenicity of *C. rogersoniana* remains limited, with only a few reports suggesting its effectiveness in controlling *Eriosoma lanigerum* [43]. In contrast, *C. rosea*, a closely related strain within the same genus, has been shown to effectively control a variety of insect pests, such as *Xylosandrus germanus* [38], *Amritodus atkinsoni* [44] and *Tenebrio molitor* [45]. Currently, *C. rosea* is widely used as a biological control agent. Given that *C. rogersoniana* belongs to the same genus, this study demonstrates its significant lethality to *Ce. chuxiongica*. As a result, *C. rogersoniana* shows great potential as a novel biological control agent, offering promising solutions for pest management.

The pathogenic process of EPF generally progresses through ten stages. Initially, fungal conidia attach to the host and begin to germinate. The hyphae then penetrate the host’s cuticle and grow within the hemocoel, producing toxins that ultimately lead to the host’s death. Following this, the hyphae invade the host’s internal organs, break through the cuticle, and generate new conidia, which spread to infect additional hosts [46,47]. In this study, scanning electron microscopy was employed to observe how Swfuy-02 infects the body wall of *Ce. chuxiongica* and to analyze the associated ultrastructural changes. The results revealed that Swfuy-02 conidia and hyphae successfully parasitize the larvae, releasing toxins that lead to their death.

Both the pathogenicity test and microscopy revealed a noteworthy observation: after inoculation with Swfuy-02 conidia suspension, hyphae and conidia initially colonized the larvae’s spiracles, a rare occurrence in fungal pest infection pathways. Over time, hyphae proliferated inside the larvae, penetrating the body wall as both conidia and hyphae, eventually covering the larvae’s head. This proliferation pattern suggests that Swfuy-02 is able to utilize the larvae’s cuticle components to establish a parasitic relationship, producing toxins that ultimately kill *Ce. chuxiongica* larvae [48,49]. These findings provide a strong basis for developing effective pest control strategies.

By identifying the optimal medium for the Swfuy-02 strain, we found that conidia production and mycelial growth were highest on Peptone Potato Dextrose Agar medium (PPDA). Compared with Sabouraud Dextrose Agar with Yeast Extract medium (SDAY) and Czapek-Dox Agar medium (CZA), this could be attributed to the presence of potatoes. Potatoes provide a rich source of starch, minerals, and vitamins, which promote enzyme synthesis and activation, improve the physical properties of the medium, and supply specific growth factors [50,51,52]. In the case of the SDAY medium, glucose serves as its primary carbon source, while the nitrogen sources are chiefly derived from peptone and yeast extract. This relatively uncomplicated nutrient combination, characterized by a high concentration (notably a relatively high glucose content), may prompt fungi to predominantly undergo vegetative growth. Specifically, this involves the growth and propagation of mycelium, rather than transitioning into the sporulation phase. Additionally, the growth and sporulation of strain depend on the activity of various enzymes, such as proteases, amylases, and cellulases. Compared with the SDAY medium, although the composition of CZA medium is relatively simple, its mineral constituents exert a crucial regulatory effect on enzyme activity. Particularly, magnesium ions, as cofactors for numerous enzymes, endow the medium with the capacity to stimulate the growth of hyphae and the functionality of active enzymes associated with sporulation.

In contrast to the Potato Dextrose Agar medium (PDA), the PPDA medium stands out with its heightened content of nitrogen sources and growth factors. This significant difference enables the PPDA medium to better meet the nutritional requirements for fungal growth and sporulation, effectively improving the sporulation efficiency of *C. rogersoniana*. Moreover, the process of fungal conidia formation is highly intricate, demanding the involvement of numerous proteins. Peptone happens to be a rich source of amino acids [53]. The amino acids contained therein serve as fundamental constituents for protein synthesis, thereby furnishing the requisite substances for the synthesis of proteins involved in fungal conidia formation. Additionally, fungal sporulation requires an adequate energy supply, and some amino acids may act as enzyme activators, maintaining their active form and accelerating metabolic reactions within the cells. This, in turn, provides the energy and materials necessary for mycelial growth and sporulation, supporting the growth and reproduction of the fungi [31,54]. Beyond amino acids, peptone may also contain small but crucial amounts of growth factors such as vitamins and nucleotides. Though required in minimal quantities, these factors play a vital regulatory role in the growth and development of strain, influencing processes like intracellular signal transduction and coenzyme synthesis and promoting both mycelial growth and sporulation [55,56,57].

In determining the median lethal time (LT_50_) and median lethal concentration (LC_50_), the Swfuy-02 strain was found to cause 50% mortality in *Ce. chuxiongica* larvae within 24.34 h at a conidia concentration of 2.35 × 10^5^ conidia/mL. This demonstrates its ability to rapidly colonize pests with a low dosage, effectively reducing the pest population before significant damage occurs. Such a rapid action is crucial for preventing large-scale pest outbreaks and the spread of infestation. Similarly, a study reported that mole crickets inoculated with a 1 × 10^7^ conidia/mL suspension of *Clonostachys* sp. experienced a 15-day mortality rate of 87% [40]. Sahin and Yanar [58] found that *C. rogersoniana* caused a 91.67% mortality rate in *Spodoptera littoralis* larvae 13 days after inoculation at a concentration of 1 × 10^8^ conidia/mL. Mahmoudi et al. [59] demonstrated that a conidial suspension at a concentration of 10^10^ conidia/mL resulted in a 46% mortality rate, assessed 7 days post-inoculation. For *C. vesuviana*, the LT_50_ and LC_50_ values were 4.6 days and 5.1 × 10^4^ conidia/mL, respectively.

In summary, a series of rigorous experimental studies and data analyses demonstrated that *C. rogersoniana* holds significant potential as a biological control agent. With the ongoing advancement of biological control technologies and the increasing demand for eco-friendly agricultural solutions, *C. rogersoniana* is poised for further development and optimization. In the future, it is expected to be widely applied in pest control across various sectors, including agriculture and forestry, making a valuable contribution to the achievement of sustainable integrated pest management goals.

### 4.2. Implications Unveiled by the Present Research

*Ce. chuxiongica* led to a decline in the output value of economic forests and disrupted the original balance of the ecosystem. The use of chemical pesticides not only increased the resistance of *Ce. chuxiongica*, but also endangered human health. In recent years, with the continuous progress of biological control technology and the increasing demand for eco-friendly agricultural solutions, there have been more and more cases of using EPF for biological control. It can not only reduce the residue of chemical pesticides in the soil and mitigate the damage to soil microbial diversity, but also reduce the risk of pesticides entering water bodies with surface runoff, which is of great significance for protecting aquatic organisms and maintaining the stability of the aquatic ecosystem, thus promoting the development of agriculture toward a more sustainable and eco-friendly direction.

In light of the manifold drawbacks of chemical pesticides, biological control approaches typified by EPF have garnered ever-growing attention. Current research shows that EPF are important biological control agents in agricultural and forestry ecosystems [60]. Many EPF, such as *Beauveria bassiana* [61], *C. rosea* [62], and *Akanthomyces muscarius* [63] have been commercially applied, and many strains also have the potential to be developed into biopesticides. However, the commercialization of *C. rogersoniana* still faces many problems. For example, *C. rogersoniana* is sensitive to the composition of the culture medium and environmental conditions, which may affect its ability to control *Ce. chuxiongica*. Therefore, in future research work, it is necessary to optimize the culture conditions of strain to ensure that *C. rogersoniana* can play a long-term and stable role and meet the needs of different regions.

Looking to the future, with the continuous development of biotechnology, there is great potential for further optimization of *C. rogersoniana*. Genetic engineering technology can be used to improve the pathogenicity and stress resistance of *C. rogersoniana* [64,65,66]. At the same time, multi-disciplinary cooperation, combining entomology, mycology, and agricultural engineering, will help to develop more scientific and effective application methods and integrated pest management strategies [67,68,69]. This will not only make *C. rogersoniana* play a greater role in pest control but also contribute to the sustainable development of agriculture.

## 5. Conclusions

In the present study, *C. rogersoniana* was successfully isolated from naturally infected cadavers of *Ce. chuxiongica*. The fungus’s ability to invade through the spiracles of *Ce. chuxiongica* significantly enhances its pathogenicity toward these pests. Our findings also revealed that, when grown on PPDA medium, this strain demonstrated the most favorable mycelial growth and sporulation efficiency. After immersing a conidia suspension of *C. rogersoniana*, the LT_50_ for *Ce. chuxiongica* larvae was determined to be 24.34 h, with an LC_50_ of 2.35 × 10^5^ conidia/mL. This study provides the first conclusive evidence that *C. rogersoniana* has a lethal effect on *Ce. chuxiongica* and holds significant potential for pest management applications, and provides a solid theoretical foundation for the development of novel pest control methods and the enhancement of pest control precision in the future. In future research endeavors, we will delve into the genes and proteins associated with the interaction between *C. rogersoniana* and *Ce. chuxiongica*, thereby analyzing the pathways of relevant substance alterations. Concurrently, optimization of the biocontrol agent will be carried out to avert harm to non-target organisms during its application.

## Figures and Tables

**Table 1:** The growth and sporulation quantity of *Clonostachys rogersoniana* on different media.

Culture Medium	Colony Diameter (mm) After 15 Days	Sporulation Quantity (×10^6^/cm^2^) After 15 Days
Potato Dextrose Agar	70.17 ± 1.2 c	3.10 ± 0.16 b
Czapek-Dox Agar	63.67 ± 1.01 d	1.24 ± 0.12 c
Peptone Potato Dextrose Agar	83.62 ± 0.97 a	19.07 ± 0.18 a
Sabouraud Dextrose Agar with Yeast Extract	71.22 ± 1.03 b	0.00 ± 0.00 d

Note: according to Two-way ANOVA and Duncan’s test (*p* < 0.05), different lowercase letters between samples indicated significant differences.

## Data Availability

Data from this trial can be found in the document and additional information package. Reagents, larvae, and microbial materials, as well as datasets used, created, and analyzed during this work are available from the reporting author upon request. The genomic sequencing data referred to in this study were stored at NCBI’s SRA database as PQ845416 (ITS) (https://www.ncbi.nlm.nih.gov/nuccore/PQ845416.1/, accessed on 7 January 2025), PV018313 (LSU) (https://www.ncbi.nlm.nih.gov/nuccore/PV018313.1/, accessed on 26 January 2025), and PQ858222 (TEF) (https://www.ncbi.nlm.nih.gov/nuccore/PQ858222.1/, accessed on 9 January 2025) and as additional sequence information. Source data are presented as additional source datasets.

## Supplementary Materials

The following supporting information can be downloaded at: https://www.mdpi.com/article/10.3390/microorganisms13040709/s1, Table S1 Medium formulation, Table S2 Primer Sets and Corresponding Amplification Targets.
