# Supplementary material for: Toxicity Assay and Pathogenic Process Analysis of Clonostachys rogersoniana Infecting Cephalcia chuxiongica
Source: Microorganisms. 2025 Mar 21;13(4):709. doi: 10.3390/microorganisms13040709 (PMC12029400; doi:10.3390/microorganisms13040709)
Supplement: Supplementary file 1 [file microorganisms-13-00709-s001.zip › microorganisms-3519201-supplementary.pdf]

Table S1 Medium formulation

Name of Medium	Formulation of Medium	Volume	Method of Sterilization
Potato Dextrose Agar	Potato 200 g; Glucose 20 g; Agar 20 g.	1 L	121℃ for 20 minutes
Czapek-Dox Agar	$NaNO_3$ 2 g; K_2HPO_4 1 g; KCL 0.5 g; $MgSO_4$ 0.5 g; $FeSO_4$ 0.01 g; Sucrose 30 g; Agar 15 g.		
Peptone Potato Dextrose Agar	Potato 200 g; Peptone 10 g; Glucose 20 g; Yeast extract 1 g; KH_2PO_4 0.5 g; $MgSO_4$ 1 g; Agar 18 g.		
Sabouraud Dextrose Agar with Yeast Extract	Yeast extract 5 g; Glucose 40 g; Peptone 10 g; Agar 20 g.		

Table S2 Primer Sets and Corresponding Amplification Targets

Target gene	Primer	Primer DNA sequence
ITS[b]	ITS1	5' TCCGTAGGTGAACCTGCGG 3'
	ITS4	5' TCCTCCGCTTATTGATATGC 3'
LSU	LR0R	5' ACCCGCTGAACTTAAGC 3'
	LR5	5' ATCCTGAGGGAAACTTC 3'
TEF	EF1-728F	5' CATCGAGAAGTTCGAGAAGG 3'
	EF2	5' GGA(G/A)GTACCAGT(G/C)ATCATGTT 3'

Clonostachys rogersoniana **GenBank ID of ITS: PQ845416**

CTGCGGAGGGATCATTACCGAGTTTACAACTCCCAAACCCATGTGAACATACCTATCGT
TGCTTCGGCGGGATCGCCCCGGGCGCCTTGCGTGCCCCGGATCCAGGCACCCGCCGGG
GGACCTTAACTCTTGTTTTATTTAGAATCTTCTGAGTAGTTTTTACAAATAAATAAAAAC
TTTCAACAACGGATCTCTTGGTTCTGGCATCGATGAAGAACGCAGCGAAATGCGAAAA
GTAATGTGAATTGCAGAATTCAGTGAATCATCGAATCTTTGAACGCACATTGCGCCCGC
CAGTATTCTGGCGGGCATGCCTGTCTGAGCGTCATTTCAACCCTCATACCCCTAGGGTG
TGGTGTTGGGGATCGGCCAAGGCCCGCAAGGGACGGCCGGCCCCTAAATCTAGTGGC
GGACCCGTCGTGGCCTCCTCTGCGAAGTAGTAATATTCCGCATCGGAGAAGCGACGAG

CCCCTGCCGTTAAACCCCCAACTTTCTAAGGTTGACCTCAGATCAGGTAGGAATACCCG
CTGAACTTAAGCATATCAATAAGCGGAGG

***Clonostachys rogersoniana* GenBank ID of LSU: PV018313**
AGCATATCAATAAGCGGAGGAAAAGAAACCAACAGGGATTGCCCTAGTAACGGCGAG
TGAAGCGGCAACAGCTCAAATTTGAAATCTGGCGCAAGCCCGAGTTGTAATTTGTAGA
GGATGTTTCTGGCGACGTGCCTTCCGAGTTCCCTGGAACGGGACGCCATAGAGGGTGA
GAGCCCCGTACGGTTGGATGCCTAGCCTCTGTGAAACTCCTTCGACGAGTCGAGTAGT
TTGGGAATGCTGCTCTAAATGGGAGGTATACGTCTTCTAAAGCTAAATACCGGCCAGAG
ACCGATAGCGCACAAGTAGAGTGATCGAAAGATGAAAAGCACTTTGAAAAGAGGGTT
AAAAAGTACGTGAAATTGTTGAAAGGGAAGCGTTCATGACCAGACTTGGGCAGGTTG
ATCATCCGTGGTTCTCCGCGGTGCACTCTGCCTGCCCAGGCCAGCATCAGTTCGCCCCG
GGGGATAAAGGTTTCGGGAATGTGGCTCCTCCGGGAGTGTTATAGCCCGTTGCGTAATA
CCCTGGGACGGACTGAGGTTCGCGCTCTGCAAGGATGCTGGCGTAATGGTCATCAACG
ACCCGTCTTGAAACACGGACCAAGGAGTCGTCTTCGTATGCGAGTGTTCGGGTGTCAA
ACCCCTACGCGTAATGAAAGTGAACGTAGGAGAGAGCTTCGGCGCATCTCCGACCGAT
CCTGATGTTCTCGGATGGATTTGAGTAAGAGCATACGGGGCCGGACCCGAAAGAAGGT
GAACTATGCCTGTATAGGGTGAAGCCAGAGGAAACTCTGGTGGAGGCTCGCAGCGGTT
CTGACGTGCAAATCGATCGTCAAATATGGGCATGGGGGCGAAAGACTAATCGAACCTT
CTAGTAGCTGGTTTCCGCCGA

***Clonostachys rogersoniana* GenBank ID of TEF: PQ858222**
CGAGAAAGTCGAGAAGGTAAGAAAACCGTCCTTTCTGCTTATCATCTGCGGGTGTTTC
GCACCTTGCCTGCCCCCTGGCAATCTGTGCCTCCATTACCCCTCCCGAAAAAAAAAAC
CTTCAGGTTTTTTTGTGGCCCATTTTAGTGGGACCACAACCCCGCCAGATTTGATAGCA
TCTCACATGAAGACACACCATGCTGACTGACTGTCTCAAAATAGGAAGCCGCCGAACT
CGGTAAGGGTTCCTTCAAGTACGCATGGGTCCTTGACAAGCTCAAGGCCGAGCGTGAG
CGTGGTATCACCATTGATATCGCTCTCTGGAAGTTCGAGACCCCCAAGTACCATGTCAC
CGTCATTGGTATGTTGTCATCTGCACGTCACCGTTCTCGTCAAAGACATGTTGGCTAAC
CATCACCATAGACGCTCCCGGTCACCGTGATTTCATCAAGAACATGATCACTGGA
